# Multiscale Distance Coherence Vector Algorithm for Content-Based Image Retrieval

**DOI:** 10.1155/2014/615973

**Published:** 2014-04-24

**Authors:** Zeng Jiexian, Liu Xiupeng, Fei Yu

**Affiliations:** ^1^School of Software, Nanchang Hangkong University, Nanchang 330063, China; ^2^School of Information Engineering, Nanchang Hangkong University, Nanchang 330063, China; ^3^School of Information Science and Engineering, Hunan University, Changsha 410082, China

## Abstract

Multiscale distance coherence vector algorithm for content-based image retrieval (CBIR) is proposed due to the same descriptor with different shapes and the shortcomings of antinoise performance of the distance coherence vector algorithm. By this algorithm, the image contour curve is evolved by Gaussian function first, and then the distance coherence vector is, respectively, extracted from the contour of the original image and evolved images. Multiscale distance coherence vector was obtained by reasonable weight distribution of the distance coherence vectors of evolved images contour. This algorithm not only is invariable to translation, rotation, and scaling transformation but also has good performance of antinoise. The experiment results show us that the algorithm has a higher recall rate and precision rate for the retrieval of images polluted by noise.

## 1. Introduction

Based on the image color, texture, shape, spatial relation, and integrating multifeatures, content-based image retrieval (CBIR) retrieves similar images and can directly find the images with specified characteristics or specific contents from the database. Shape, as one of the most important image features, is an important basis for people to understand images and also is more in line with people's visual perception. Shape-based image retrieval has become a hot topic for researches. Approaches for shape representation and retrieval can be broadly classified into contour-based and region-based. The methods of the contour-based are mainly chain code [1]. Polygonal approximation, Fourier descriptors [2, 3], wavelet descriptors, scale space [4, 5], and so forth. And some of the region-based methods are mainly geometric moment invariants, orthogonal moments [6, 7], and so on.

In terms of contour-based image retrieval, distance histogram [8] is a method commonly applied for shape description, but it cannot reflect the spatial distribution of contour pixels and different shapes may be with the same distance histogram; so it will lead to a higher false retrieval rate. To address this problem, [9] proposed the concept of distance coherence vector by the analogy with the concept of color coherence vector, which is based on the distance histogram; the pixels within the bucket are divided into coherent ones and incoherent ones according to certain criterion, which makes up for parts of the shortcomings of the distance histogram. However, there is still the same distance coherence vector for different shapes. An improved distance coherence vector algorithm was proposed in [10]. The centroid distance of the average coordinates for the biggest connected coherence pixels of image contours is introduced into distance coherence vector as a new feature, which improves the ability to distinguish different shapes. However, the problem that different shapes are with the same descriptor still exists. Besides, the image retrieval algorithm based on distance coherence vector has not achieved ideal effect on retrieving those images polluted by noise; that is, it is with relatively poorer antinoise performance.

Shape descriptors based on scale space are also often used for image retrieval and have a good performance of antinoise. In [4, 5], the method of retrieving image by using curvature scale space descriptor is applied. Curvature scale space descriptor is a closed contour descriptor used by MPEG-7 standard image database and has performance of translation, rotation, and scale invariance, as well as relatively stronger noise immunity. In [11], a contour-based image retrieval algorithm is proposed. It firstly evolves the image contour curve and then the descriptor is constructed by using distance histogram between the evolutionary contour curve and the skeleton. The descriptor has comparatively stronger noise immunity but does not reflect the spatial distribution feature of contour points.

In order to solve the problems on different shapes with the same descriptor and poor performance of antinoise by distance coherence vector algorithm for image retrieval, this paper proposes a multiscale distance coherence vector algorithm which not only is invariable to translation, rotation, and scaling by normalization but also has better noise immunity. In this paper, the descriptor used for image retrieval which was obtained by reasonable weight distribution of the distance coherence vectors of contour curves with different scales can achieve a better retrieved result than the original algorithm. The comparative experiment results show us the effectiveness and robustness of this algorithm.

## 2. The Shortcomings of Distance Histogram and Distance and Distance Coherence Vector

### 2.1. Different Shapes Have the Same Descriptor

The centroid distance refers to the distance between the contour point and its centroid, including the information of the shapes and is commonly used for shape description. Fan [8] uses the centroid distance histogram (DH) to describe shapes. The centroid distance histogram is defined as the number of contour points quantified to certain bucket from centroid distance as follows. In *H* : (*d*_0_, *d*_1_,…, *d*_*N*−1_), where *N* is the number of buckets in the histogram, *d*_*i*_  (*i* = 0,1,…, *N* − 1) is the corresponding number of contour points quantified to the certain bucket. The DH is invariable to translation, rotation and scaling by normalization. Although the DH is an effective method of shape description, different shapes still have the same or similar distance histogram. Sajjanhar proposed the concept of distance coherence vector (DCV) by analogy with the method of color coherence vector; that is, in accordance with certain criterion, the pixels within each bucket are divided into coherent and incoherent ones. Supposing that there are *S* or over *S* consecutive sample points which belong to the same bucket, thus, the pixels are called coherent pixels, otherwise called incoherent pixels. Here, *S* is the threshold value, when *S* = 1; the distance coherence vector degenerates into distance histogram.

We consider the distance histogram of certain contour as *H* : [*α*_0_, *α*_1_,…, *α*_*N*−1_] and centroid distance coherence vector as $\tilde{H}:\left[\left(\alpha_{0},\beta_{0}\right),\left(\alpha_{1},\beta_{1}\right),\ldots ,\left(\alpha_{N-1},\beta_{N-1}\right)\right]$.

**Figure 1:**
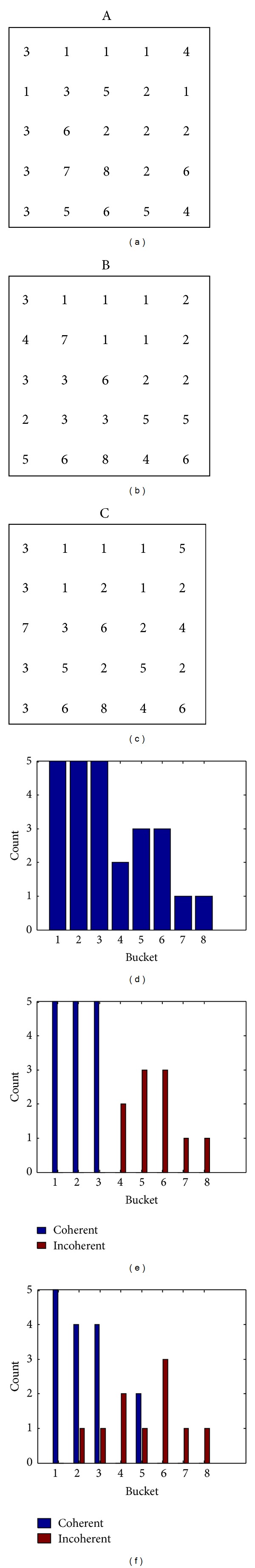
Centroid distance matrices and their DH, DCV histogram of 3 images. (a) Centroid distance matrix of image A; (b) centroid distance matrix of image B; (c) centroid distance matrix of image C; (d) DH of images A, B, and C; (e) DCV histogram of images A and C; (f) DCV histogram of image B.

Here, we give an example of the centroid distance matrices of different images and their corresponding histograms. As shown in Figures 1(a), 1(b), and 1(c), there are the centroid distance matrices of three different images while their DH and DCV histogram are, respectively, presented in Figures 1(d) and 1(e). It can be seen from the figures that images A, B, and C have the same DH and images A and C have the same DCV histogram. By assuming that the threshold value of the coherent pixels number is 3, the DCV of images A and B are respectively as follows:

(1) $$\begin{array}{c} {\tilde{H}}_{\text{A}}:\left[\left(5,0\right),\left(5,0\right),\left(5,0\right),\left(0,2\right),\left(0,3\right),\left(0,3\right),\left(0,1\right),\left(0,1\right)\right],\\ {\tilde{H}}_{\text{B}}:\left[\left(5,0\right),\left(4,1\right),\left(4,1\right),\left(0,2\right),\left(2,1\right),\left(0,3\right),\left(0,1\right),\left(0,1\right)\right]. \end{array}$$

It can be seen that the images A and B have the same DH but are with different DCV. This shows us that DCV has better ability to describe the differentiation of shapes. Although DCV has better ability than DH to distinguish shapes, different images may still have the same DCV. For example, the DCV of image C is as follows:

(2) $$\begin{array}{c} {\tilde{H}}_{\text{C}}:\left[\left(5,0\right),\left(5,0\right),\left(5,0\right),\left(0,2\right),\left(0,3\right),\left(0,3\right),\left(0,1\right),\left(0,1\right)\right]. \end{array}$$

Obviously, it is the same with the DCV of image A; thus, although DCV has better ability to distinguish shapes, it cannot fully distinguish images of different shapes. In [10], an improved distance coherence vector (IDCV) was proposed in accordance with the problem that different shapes are still with the same DCV. The idea is finding the biggest connected coherence pixels in each bucket in the process of calculating DCV and calculating its average coordinate. The distance (called ND) between the average coordinate and the centroid is calculated and added to shape feature by normalization. The similarity of traditional DCV and that of newly added feature vector (ND) are calculated by different distance measurement methods. Besides, the final similarity index of image will be obtained by integrating the similarity of those two subfeatures. The IDCV algorithm improves the ability to distinguish shapes, but different shapes still have the same IDCV. For example, the average coordinate of images A and C of the biggest connected coherence pixels are as follows: [(1.4,3), (3,4), (3,1.2), (0,0), (0,0), (0,0), (0,0)]. By assuming that the centroid coordinates of images A and C are the same, thus the average coordinate of the biggest connected coherence pixels and new features determined by centroid are the same in IDCV algorithm. Besides, the centroid distance may be the same even if the average coordinates are different. By assuming that the average coordinates of the biggest connected coherence pixels of two images are, respectively, [(2, 3), (1, 7), (0, 0)], [(5, 4), (6, 0), (0, 0)] and their centroid coordinates are (4, 3) and (3, 4), respectively, then their centroid distance is (2, 5, 5). It can be seen from the above analysis that the distinguishing ability of DCV is better than DH but worse than IDCV. However, different shapes may still have the same IDCV.

### 2.2. The Shortcomings of Antinoise Performance

**Figure 2:**
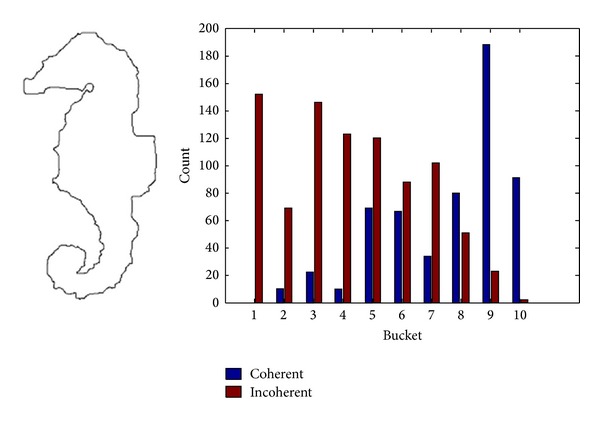
Hippocampus image in the SQUID and its corresponding DCV histogram.

**Figure 3:**
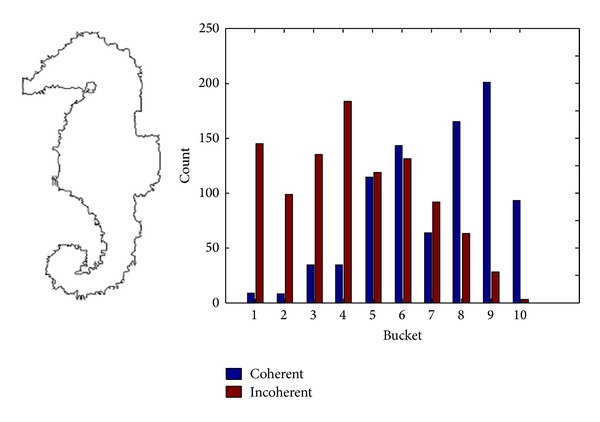
Hippocampus image polluted by noise and its corresponding DCV histogram.

It is found in the experiment that image retrieval algorithm based on DCV is sensitive to noise.  Figure 2 shows the Hippocampus image in the SQUID standard test database and its corresponding DCV histogram, while Figure 3 shows the Hippocampus image polluted by noise and its corresponding DCV histogram. It can be seen that there are great differences between the DCV of those two images that will affect the retrieval performance. Hence, for images polluted by noise, relatively higher false retrieval rate will be resulted in by adopting the DCV algorithm or IDCV algorithm. The similarity of those two images is calculated for DCV algorithm by methods presented in [10] as follows:

(3) $$\begin{array}{c} D_{\text{DCV}}=\overset{N-1}{\underset{i=0}{\sum }}\left|\alpha_{Qi}-\alpha_{Ii}\right|+\left|\beta_{Qi}-\beta_{Ii}\right|. \end{array}$$

And the distance of the newly added feature namely ND in IDCV algorithm is calculated as follows:

(4) $$\begin{array}{c} D_{\text{ND}}={\left(\overset{N-1}{\underset{i=0}{\sum }}{\left|ND_{Qi}-ND_{Ii}\right|}^{2}\right)}^{0.5}. \end{array}$$

The similarity of two images for IDCV algorithm is defined as

(5) $$\begin{array}{c} D_{\text{IDCV}}=\omega \cdot D_{\text{DCV}}+\left(1-\omega \right)\cdot D_{\text{ND}}, \end{array}$$

where *α*_*Qi*_ and *β*_*Qi*_ are respectively the coherent pixels and incoherent pixels in the *i*th bucket of DCV descriptor in original image as shown in Figure 2. Similarly, *α*_*Ii*_ and *β*_*Ii*_ are, respectively, coherent pixels and incoherent pixels of DCV descriptor in image polluted by noise as shown in Figure 3. ND is a newly added feature of IDCV algorithm, namely, the centroid distance of the biggest connected coherence pixels.

The similarity of two images for DCV algorithm computed from (3) is *D*_DCV_ = 558. The similarity of two images for IDCV algorithm computed from (5) is *D*_IDCV_ = 442.6, where the value of *ω* is 0.7.

It can be seen from *D*_DCV_ = 558 and *D*_IDCV_ = 442.6 that the difference of similar images polluted by noise adopting the DCV or IDCV algorithm is comparatively larger; thus DCV and IDCV algorithms are with poorer performance of antinoise.

## 3. Structure of Multiscale Distance Coherence Vector Descriptor

As the image retrieval algorithm based on DCV or IDCV is relatively more sensitive to noise, a higher false retrieval rate will be resulted in by image retrieval for the image database seriously polluted by noise. This paper proposes an image retrieval algorithm of multiscale distance coherence vector in order to reduce the impact of noise. The basic idea of the algorithm is as follows: firstly evolve images contour with different scale factors and then obtain multiscale distance coherence vector by integrating the distance coherence vector of the evolved images contour. The descriptor used for image retrieval can effectively reduce the impact resulted from noise.

### 3.1. The Evolution of Contour Curve

For contour-based image retrieval, the contour shape can be obtained by edge detection or image segmentation, while the contour gotten here is often severely affected by noise. In order to construct shape descriptor with relatively stronger robustness, this paper evolves the contour curve with Gaussian function. After the evolution, not only the impact of noise is reduced, but also the contour curve is more in line with the human visual perception [12]. Suppose that the parametric vector equation for a contour curve is as follows:

(6) $$\begin{array}{c} r\left(u\right)=\left(x\left(u\right),y\left(u\right)\right), \end{array}$$

where *u* is an arbitrary parameter. The process of curve evolution using 1D Gaussian function to convolve contour curves is as

(7) r(u,σ)=(X(u,σ),Y(u,σ))=r(u)∗g(u,σ)=(x(u)∗g(u,σ),y(u)∗g(u,σ)),

where *r*(*u*, *σ*) is the evolved curve and ∗ is convolution operation. $g\left(u,\sigma \right)=\left(1/\sqrt{2\pi }\sigma \right)e^{-u^{2}/2\sigma^{2}}$ is a 1D Gaussian function, the kernel of which is *σ*. And in the process of evolution *σ* is defined as scale factor.

In order to facilitate the description for process of convolution, the formula for computing the curvature of each point in the curve can be expressed as

(8) $$\begin{array}{c} k\left(u,\sigma \right)=\frac{\left|X_{u}\left(u,\sigma \right)Y_{uu}\left(u,\sigma \right)-X_{uu}\left(u,\sigma \right)Y_{u}\left(u,\sigma \right)\right|}{{\left(X_{u}{\left(u,\sigma \right)}^{2}+Y_{u}{\left(u,\sigma \right)}^{2}\right)}^{1.5}}. \end{array}$$

The first derivatives of contour curve coordinates can be calculated by

(9) $$\begin{array}{c} X_{u}\left(u,\sigma \right)=x\left(u\right)*g_{u}\left(u,\sigma \right);\\ X_{uu}\left(u,\sigma \right)=x\left(u\right)*g_{uu}\left(u,\sigma \right). \end{array}$$

And the formula for computing the second derivatives of contour curve coordinates is similar for *Y*_*u*_(*u*, *σ*) and *Y*_*uu*_(*u*, *σ*).

**Figure 4:**
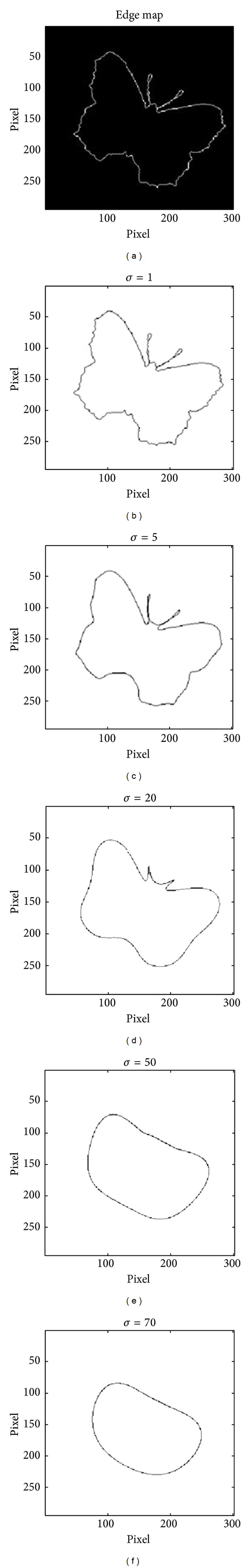
The evolution of image contour curve. (a) Original image; (b) the evolution of contour curve when *σ* = 1; (c) the evolution of contour curve when *σ* = 5; (d) the evolution of contour curve when *σ* = 20; (e) the evolution of contour curve when *σ* = 50; (f) the evolution of contour curve when *σ* = 70.

The Gaussian convolution of contour curve is essentially a weighted average of contour points. Gaussian function assigns different values to all contour points. By assuming that *P*_0_ is the central point, then the weight value is the largest and the weight value of the surrounding contour points will gradually decrease until being close to zero as the distance from the central point increases. From the perspective of the curvature, the function of contour curve convolving with Gaussian is having the place where the curvature zero crossing of the contour curve continually moves towards the convexity part and the curvature of the concavity part is gradually close to zero and the curvature of the noise points in the contour is slowly close to the curvature of the nonnoise points, so as to make the curve smooth.  Figure 4 shows us one image in the MPEG-7 image database and its evolution result of the contour curve with different scale factors.

It can be seen from Figure 4 that the contour curve becomes smoother and smoother with the increase of *σ*, namely, the scale factor. In other words, the contour with large scale removes most noise and local information and only retains the overall information of the contour, while the contour with small scale retains more local information and noise. From Figures 4(e) and 4(f), we can see that with the increase of  *σ*, the contour curve gradually evolves into a shape similar to an oval and seriously becomes distorted when *σ* increases to certain extent, so the scale factor should not be too large. The criterion for choosing the value of scale factor is maintaining the shape of the contour itself while removing the noise at the same time. The image retrieval performance for the images suffering from noise can be enhanced by applying descriptor of contour integrating with different scales.

### 3.2. Implementation Steps of Multiscale Distance Coherence Vector

It illustrates that the DCV and IDCV algorithms are sensitive to noise in Section 2.2 and different shapes may have the same descriptor. In order to further improve the ability of DCV algorithm to distinguish different shapes as well as the noise immunity, this paper proposes a multiscale distance coherence vector (MDCV) which is used to describe shapes. Multiscale distance coherence vector algorithm adopts Gaussian function to evolve contour curve and the impact of noise is effectively reduced. The algorithm has the multiscale method applied in DCV, which can give a better solution to those two problems described in Section 2. It can be seen from Figure 1 that although Figures 1(a) and 1(c) are with the same DCV, their centroid distance has different spatial distribution. The essence of curve evolved by Gaussian function is a weighted average of the curve points. So there will be different descriptors for evolved contour curves which are with different spatial distribution of centroid distance and can better reduce the impact of noise. The implementation steps of MDCV algorithm are as follows.

**Step 1**

The image contour *I*(*x*, *y*) is obtained through contour tracking algorithm and then the centroid distance matrix *D*(*x*, *y*) is calculated. The centroid distance of the contour point *i*(*x*, *y*) is computed as follows:

(10) $$\begin{array}{c} d\left(i\right)=\sqrt{{\left(x_{i}-x_{0}\right)}^{2}+{\left(y_{i}-y_{0}\right)}^{2}}, \end{array}$$

where (*x*_0_, *y*_0_) is the centroid coordinate of the contour and *M* is the pixels number in the contour. The centroid coordinate is computed as shown below:

(11) $$\begin{array}{c} x_{0}=\frac{1}{M}\overset{M-1}{\underset{i=0}{\sum }}x_{i},\;\;y_{0}=\frac{1}{M}\overset{M-1}{\underset{i=0}{\sum }}y_{i}. \end{array}$$

Then, the centroid distance matrix of original image *D* ^(1)^(*x*, *y*) is calculated.

**Step 2**

The Gaussian function with two different scale factors *σ*_1_ and *σ*_2_ are, respectively, used to evolve original image contour *I*(*x*, *y*) to obtain *I*_1_(*x*, *y*) and *I*_2_(*x*, *y*), which are the evolved contours. The centroid distance matrices of evolved contour curves *D* ^(2)^(*x*, *y*) and *D* ^(3)^(*x*, *y*) are calculated with the same method as Step 1, where scale factors are, respectively, *σ*_1_ = 5 and *σ*_2_ = 20.

**Step 3**

Normalized centroid distance matrix: the matrices obtained by the first two steps are discrete numerical sets and all elements in the centroid distance matrices are normalized within the range of [1, *N*_bin_], in order to let centroid distance matrix be with scaling invariance. The normalization formula is shown as follows:

(12) $$\begin{array}{c} d_{\text{normal}}=\frac{d}{d_{\max}}\times N_{\text{bin}}, \end{array}$$

where *d* is the elements in the centroid distance matrix and the range is [*d*_min⁡_, *d*_max⁡_], in which *d*_max⁡_ is the maximum value and *d*_min⁡_ is the minimum value of the matrix. *N*_bin_ is the series of distance histogram. *d*_normal_ is the normalized value, the range of which is [1, *N*_bin_].

**Step 4**

**Figure 5:**
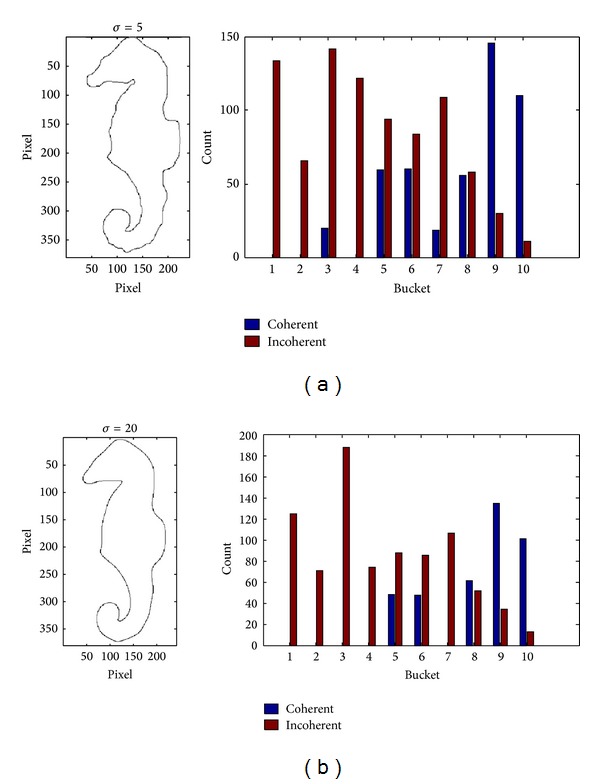
The evolved contour curves and their DCV histograms of the original image with different scale factors. (a) *σ* = 5; (b) *σ* = 20.

Calculated DCV: find connected pixels within each bucket by using eight-neighborhood searching method and label those pixels which are greater than the threshold value *κ* as coherent pixels, or else as incoherent pixels, so the elements in centroid distance matrix are divided into coherent ones and incoherent ones. The number of coherent pixels and incoherent pixels are further counted for all the buckets.  Figure 5 shows us the evolved contour curve and its MDCV histogram with different scale factors for the Hippocampus image of the Figure 2.

**Figure 6:**
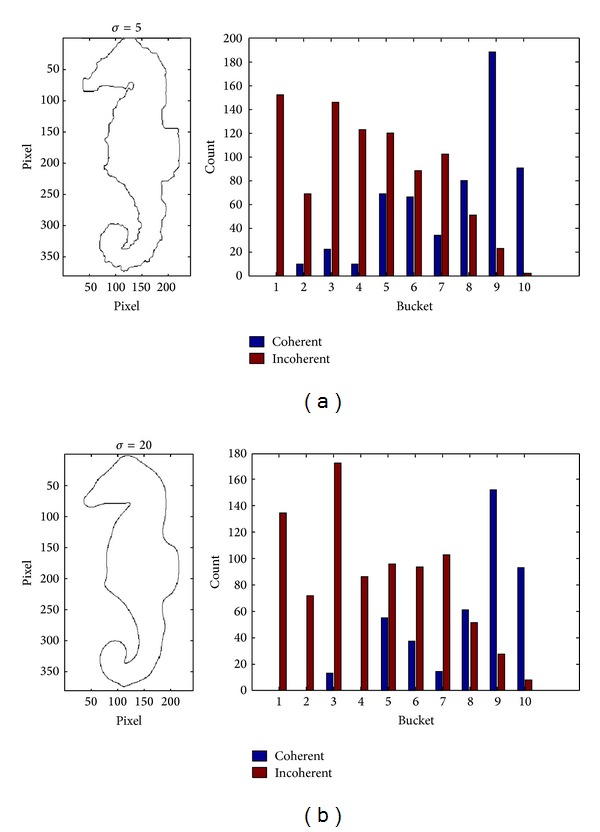
The evolved contour curves and their MDCV histograms with different scale factors for the image polluted by noise. (a) *σ* = 5; (b) *σ* = 20.

**Figure 7:**
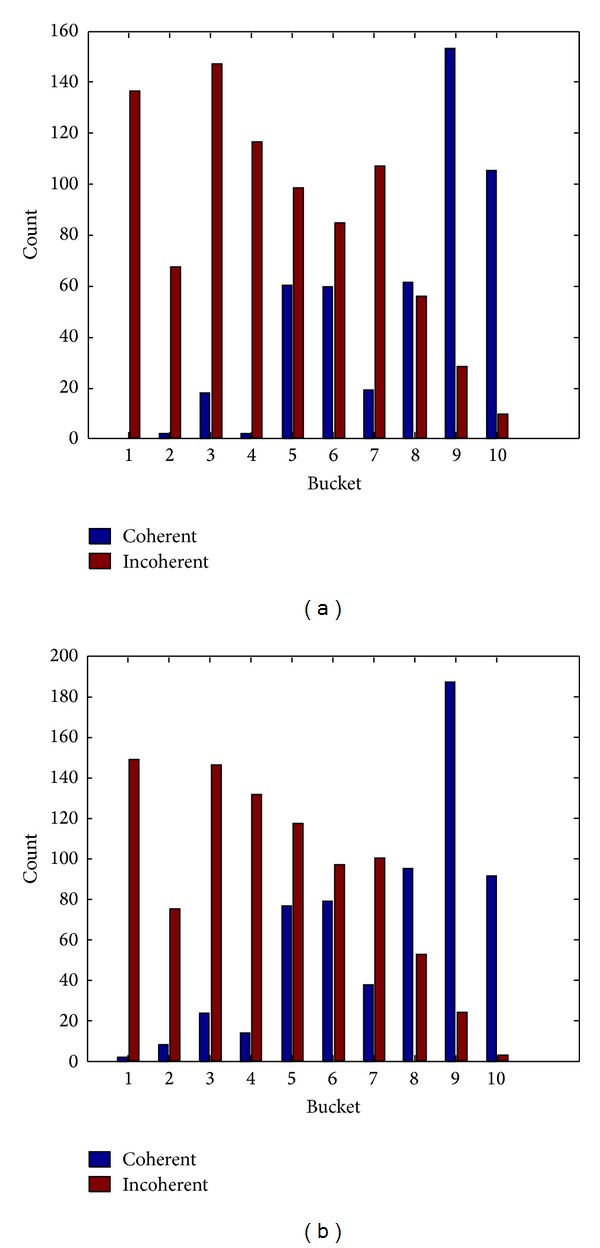
The MDCV histogram of two images. (a) The MDCV histogram of original Hippocampus image; (b) the MDCV histogram of Hippocampus image polluted by noise.

Figure 6 shows us the evolved contour curve and MDCV histogram with different scales for the Hippocampus image which is polluted by the noise in Figures 3 and 7 shows the MDCV histogram integrated according to different weights of DCV of evolved contour curves with different scales. The similarity of Figures 7(a) and 7(b) for IDCV algorithm is computed from (3) as follows:

(13) $$\begin{array}{c} D_{\text{MDCV}}=228.1. \end{array}$$

It can be seen from those distances of similar images, namely, *D*_DCV_, *D*_IDCV_ and *D*_MDCV_, that MDCV can effectively reduce the impact of noise.

## 4. Similarity Measurements

In terms of the algorithm in this paper, the MDCV can be obtained by integrating the DCV of contour with three different scales which are the original image and two evolved images with the scale factors *σ*_1_ and *σ*_2_.

By considering that *I* is an image to be retrieved and *D* ^(1)^(*x*, *y*) is its DCV, *D* ^(2)^(*x*, *y*) and *D* ^(3)^(*x*, *y*)  are the DCV of the evolved images. Thus, the MDCV of the image is computed as follows:

(14) $$\begin{array}{c} D\left(I\right)=\omega_{1}\cdot D^{\left(1\right)}\left(I\right)+\omega_{2}\cdot D^{\left(2\right)}\left(I\right)+\left(1-\omega_{1}-\omega_{2}\right)\cdot D^{\left(3\right)}\left(I\right), \end{array}$$

where *ω*_1_ and *ω*_2_ are the weight values, the range of which is (0, 1) and *D*(*I*) is the MDCV of image. The similarity of images is calculated by distance criterion. Here, the superscript indicates the scale sequence but not the actual value of scale factors. By assuming that *I* is the image to be retrieved, while *Q* is the image database and the series of the MDCV is *N*_bin_, then the distance between image *I* and images in image database is computed as follows:

(15) $$\begin{array}{c} D\left(Q,I\right)=\overset{N_{\text{bin}}-1}{\underset{i=0}{\sum }}\left|\alpha_{Ii}-\alpha_{Qi}\right|+\left|\beta_{Ii}-\beta_{Qi}\right|. \end{array}$$

## 5. Analysis of Algorithm Performance

In order to test the effectiveness and noise immunity of the algorithm in this paper, the images in the experiment include rotation and scaling transformation and noise superimposition. The performance of the algorithm will be demonstrated from the following three aspects.

### 5.1. The Performance of the Algorithm under Changes in Rotation

**Figure 8:**
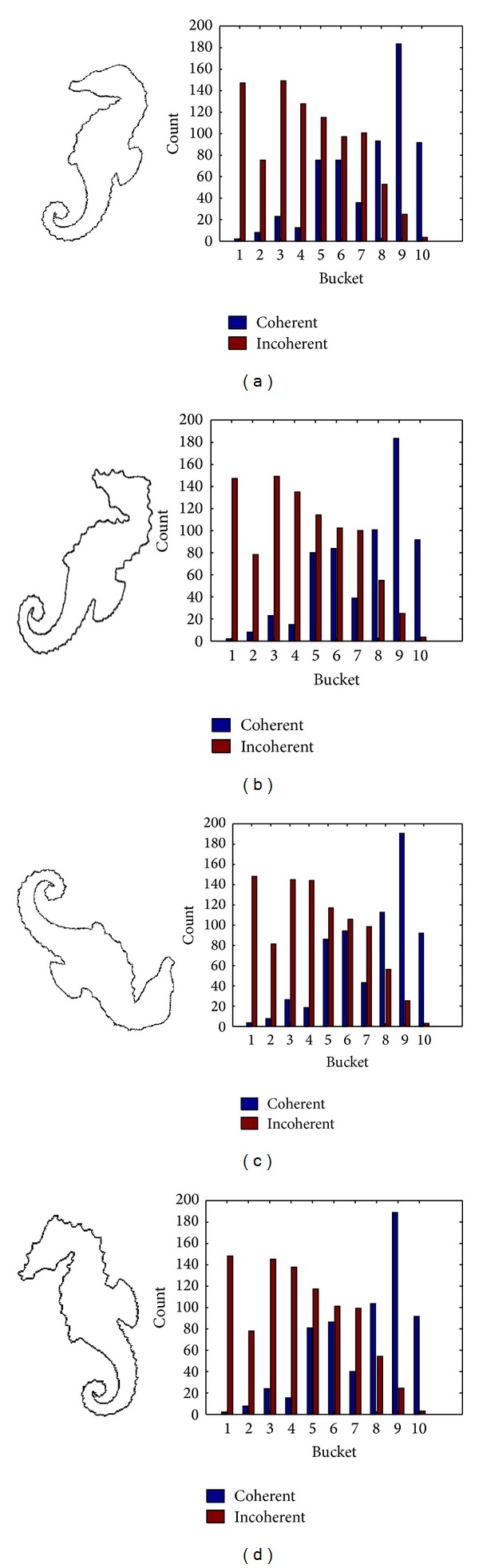
The MDCV histogram of Hippocampus images after rotation in different angles; (a) clockwise rotation through 20 degrees; (b) clockwise rotation through 60 degrees; (c) clockwise rotation through 120 degrees; (d) clockwise rotation with 330 degrees.

Figure 8 shows us the Hippocampus images after rotation in different angles and their corresponding MDCV histogram. It can be seen from the figures that the images after rotation are with very similar histograms and the distances between original image and the image after rotation which are calculated by (15) are, respectively, as follows:

(16) $$\begin{array}{c} D_{1}=38.5,\;D_{2}=64.1,\;D_{3}=48.4,\;D_{4}=52.9. \end{array}$$

This shows us that the algorithm is invariable to rotation because both distance coherence vector and evolution of the curve have nothing to do with orientation.

### 5.2. The Algorithm Performance of Scaling Transformation

**Figure 9:**
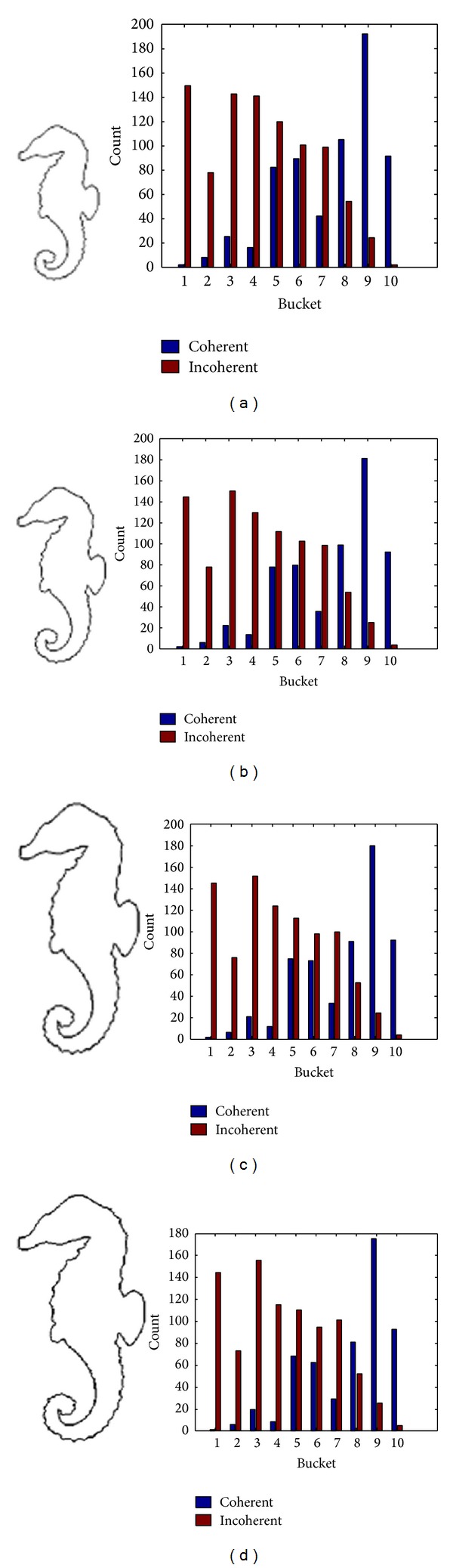
The MDCV histogram of Hippocampus images after scaling transformation. (a) Image scaled down 0.5 times; (b) image scaled down 0.8 times; (c) image scaled up 1.2 times; (d) image scaled up 1.5 times.

Figure 9 shows the Hippocampus images after scaling transformation and their corresponding MDCV histograms. It can be seen from the figures that images after scaling transformation are with very similar MDCV histograms. The distance between the original image and the images after scaling transformation are shown as

(17) $$\begin{array}{c} D_{1}=55.2,\;D_{2}=62.4,\;D_{3}=49.6,\;D_{4}=50.3. \end{array}$$

This shows that the algorithm is invariable to scales mainly due to the normalization in the process of forming centroid distance matrix.

### 5.3. Antinoise Performance of Algorithms

**Figure 10:**
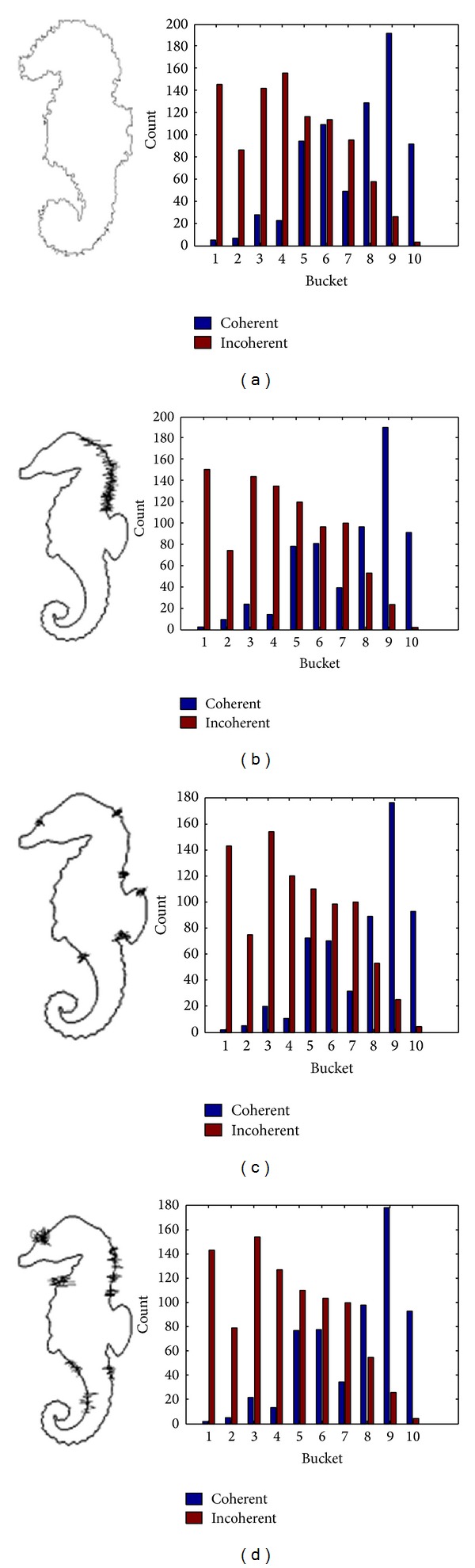
The MDCV histogram of Hippocampus images polluted by different degree noise. (a) Image added noise 1; (b) image added noise 2; (c) image added noise 3; (d) image added noise 4.

Figure 10 shows the MDCV histograms of Hippocampus images polluted by different degree noise. It can be seen from the figures that those images are with similar MDCV histograms. The MDCV distance between original image and the images polluted by noise are shown as follows:

(18) $$\begin{array}{c} D_{1}=217.2,\;D_{2}=185.7,\;D_{3}=168.5,\;D_{4}=202.3. \end{array}$$

This shows us that the algorithm has good noise immunity, which is mainly because the impact of different degree noise added to the contour curve is effectively reduced by integrating DCV of contour curves after evolution by means of Gaussian function to get comparatively smoother curves.

## 6. Experimental Results and Analysis

### 6.1. Experiment 1

Experiment 1 applies the MPEG-7 image test database which is widely used for image retrieval. We select 20 categories of images with significant differences. And each type is with 20 extended images and contains displacement, rotation, and scale transformation. Let these 400 images be an image database. In Table 2, there are some query images. In the experiment, “recall rate” and “precision rate” are adopted as algorithm evaluation criterion for retrieving results. Under the condition when the recall rate is the same, the higher the precision rate is, the better the retrieval results of this algorithm is. To illustrate the superiority of the algorithm, a comparison is made among the MDCV algorithm and DCV and IDCV algorithms.

We adopt “precision rate” and “recall rate” as algorithm evaluation criterion for retrieval results in the experiments. Consider

(19) $$\begin{array}{c} \text{Precision}=\frac{N_{\text{correct}}}{N_{\text{retrieve}}},\\ \text{Recall}=\frac{N_{\text{correct}}}{N_{\text{corrlate}}}, \end{array}$$

where *N*_correct_ is the number of retrieved images which are related to query images, *N*_retrieve_ is the number of retrieved images, and *N*_corrlate_ is the number of all the images which are related to query images in the image database.

**Figure 11:**
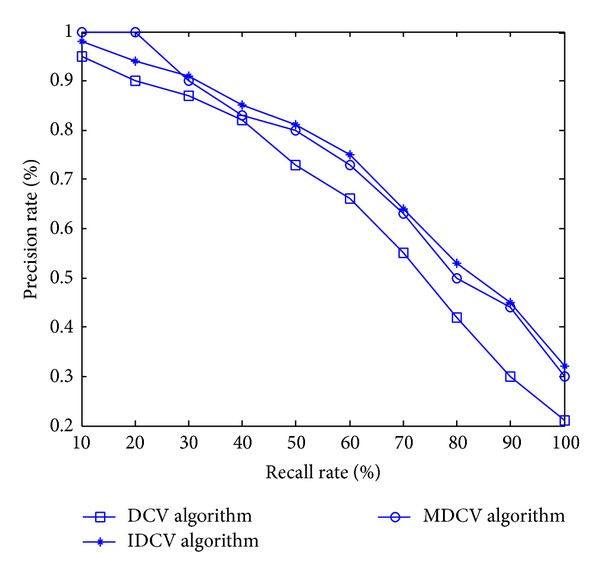
Contrast curves of DCV, IDCV, and MDCV algorithms.

Five categories of images in the image database (as shown in Table 2) are selected as query images for the experiment. The centroid distance is quantified to 10 buckets. Consider the threshold value *S* = 7 and the scale factors *σ*_1_ = 5, *σ*_2_ = 20, and *ω*_1_ = 0.2, *ω*_2_ = 0.6.  Figure 11 shows us the contrast curves for the precision rate and recall rate for IDCV, DCV, and MDCV algorithms. The value of curves is the average value of the precision rate and recall rate based on these five categories of images. It can be seen from the contrast curves that the retrieval effect achieved by MDCV algorithm is better than that by DCV, while slightly worse than what IDCV achieved, but the recall rate is higher than IDCV algorithm when the precision rate is high.

### 6.2. Experiment 2

**Figure 12:**
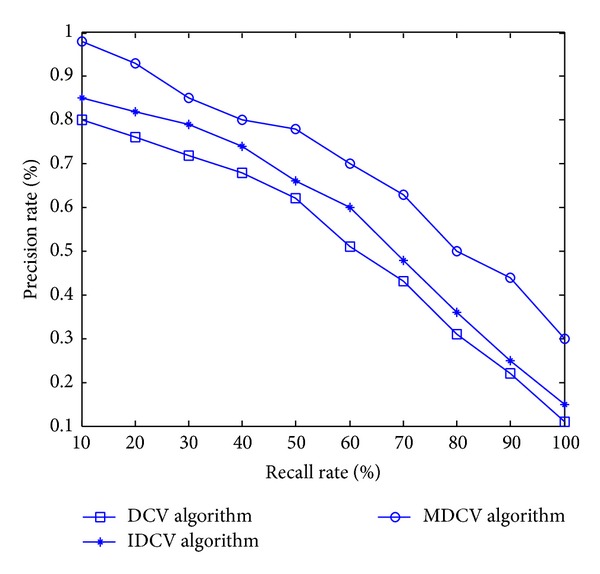
Contrast curves of DCV, IDCV, and MDCV algorithms.

We adopt SQUID standard test database in Experiment 2 and select 10 categories of images with significant differences. Each categories contains 20 extended images, the 20 extended images are obtained by arbitrary angle rotation transformation, 0.5–1.5 times scale transformation and random noise pollution from the original image. Therefore, we build a image database which containing 200 images.  Table 1 shows us five images selected from the image database. In the experiment, by assuming that the scale factors *σ*_1_ = 5 and *σ*_2_ = 20 and *ω*_1_ = 0.2 and *ω*_2_ = 0.7, here, we still compare MDCV with DCV and IDCV algorithms.  Figure 12 is the contrast curves of these three algorithms. The value of curves is the average value of the precision rate and recall rate for those five categories of images.

It can be seen from the contrast curves of this experiment that MDCV algorithm is obviously superior to DCV and IDCV algorithms for retrieval of the image database which is badly polluted by noise.

### 6.3. Experiment Summary

It can be seen from the above two experiments that, in terms of MPEG-7 image database, the retrieve effect of MDCV algorithm is slightly worse than that of IDCV algorithm, but better than that of DCV algorithm. Besides, in the premise of high precision rate, the recall rate of MDCV is higher than that of IDCV algorithm, which indicates that MDCV algorithm can be used for ordinary image retrieval. In the SQUID image database and the DCV and IDCV algorithms cannot achieve relatively better retrieval results because of noise, while MDCV algorithm still achieves comparatively more ideal results which are better than those of DCV and IDCV algorithms. It shows us that MDCV algorithm has better performance of antinoise and could be used for the retrieval for those images polluted by noise.

### 6.4. Retrieval Time

As the algorithm firstly evolves contour and then extracts DCV from the evolved contour, the retrieval of this algorithm lasts longer. All requisite experiment are implemented in Matlab. And the system operating environment is Windows XP, CPU 2.66 GHz, memory 2 G. Taking MPEG-7 image database for example, Table 3 show us the retrieval time of those three algorithms.

## 7. Conclusions

This paper proposes a multiscale distance coherence vector algorithm in accordance with problems that different shapes are with the same descriptor and poor performance of antinoise of the image retrieval algorithm based on DCV. Firstly, this algorithm obtains relatively smoother contour curves by Gaussian function evolving contour curve. Then, respectively, calculate DCV of the original contour curve and evolved contour curves. The multiscale distance coherence vector is finally obtained by reasonable weight distribution of DCV of contour curves with different scales. This algorithm not only is invariable to translation, rotation, and scaling transformation, but also has the good performance of antinoise. In the comparison experiment for image database polluted by noise, MDCV algorithm has a higher recall and precision rate than those of DCV and IDCV algorithms, but the retrieval lasts longer.

## Figures and Tables

**Table 1:** Some query images in the MPEG-7 image database.

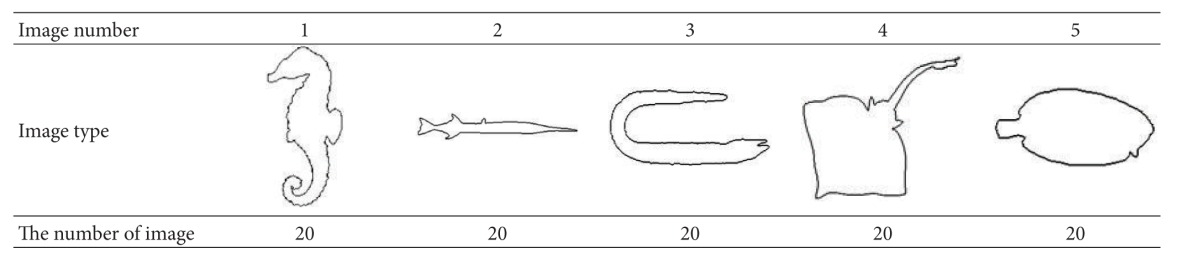

**Table 2:** Some images in image database of SQUID.

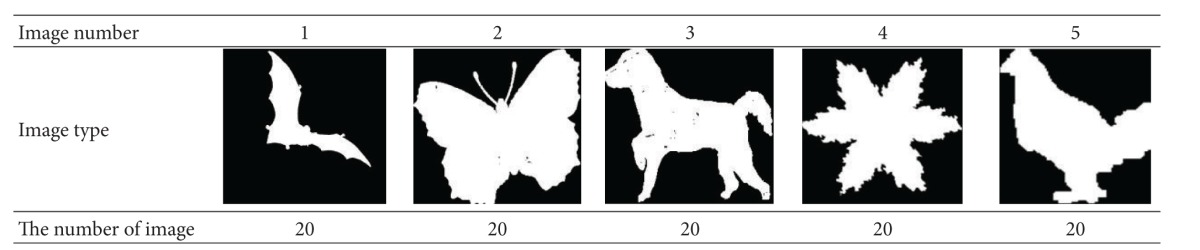

**Table 3:** The comparison of retrieval time among MDCV, DCV, and IDCV algorithms.

Algorithm	DCV	IDCV	MDCV

Relative time (s)	51.3180	58.8875	63.4581
